# Some Notes on Common Skin Diseases

**Published:** 1906-09-08

**Authors:** 


### SOME NOTES ON COMMON SKIN DISEASES.

### Diet.

Diet in skin diseases is a subject on which dermatologists differ considerably in their opinions. While with some the limitation of certain articles of diet and the prohibition of others is a matter of primary importance, results, it must be assumed, quite as satisfactory are obtained by others who are content with simple directions only. Malcolm Morris [1] goes so far as to say that: " It is in comparatively few cases of skin disease that the diet is really of any particular importance. The guiding principle is to forbid anything that is likely to aggravate the existing disease by overstimulating or otherwise disordering the local circulation, or to produce fresh lesions by causing reflex disturbances or toxæmia." He is content, as a rule, to leave the choice of diet to the patient himself, trusting that his common sense will teach him to avoid what is deleterious. There are, without doubt, certain patients who show a predisposition to erythema or urticaria, etc., with certain articles of diet. Among these may be mentioned shellfish, pork, mushrooms, certain vegetables, such as parsley, and fruits such as strawberries. These seem to act in a similar way to the toxins in ptomaine poisoning, serum injections, or constipation. There are drugs also which in their elimination through the skin may produce eruptions. These conditions, depending as they do upon definite factors, are evanescent in character. Alcohol is well known to produce a flushing of the skin and hence is responsible for aggravating an existing skin lesion and especially for increasing a pruritus. Hot condiments and tobacco cause flushing, and coffee produces itching. But with these matters of common knowledge we may follow Walter Smith in his conclusions that: "(1) Very few skin diseases are directly traceable to dietetic causes, but improper diet may aggravate existing eruptions. Idiosyncrasy must be largely allowed for. (2) The diseases that may so arise are of a transitory character, and mostly belong to the class of erythemata. (3) Diet has very little influence in promoting the cure of cutaneous eruptions. The results are far behind popular expectations, even in such cases as acne rosacea, where we are led to hope for much. (4) Avoidance of alcohol, regulation of the bowels, and cure of anæmia are of infinitely greater importance than special dieting in the management of skin diseases." In special conditions some indication may be found. In eczema, for instance, Besnier is of the opinion that diet is of great importance. He considers that most patients "eat too much or too well out of proportion to the combustion performed." Brocq believes the regulation of the diet to be the most efficacious internal treatment of eczema. Morris, however, does not consider that any particular dietetic restriction is called for in chronic eczema (except as to alcohol), and in acute or very severe cases a simple diet, or milk only for a time, is indicated. Jamieson [2] is practically of the same opinion, considering that the diet needs regulation, though more in the direction of being plain and varied than in the institution of vexatious restrictions. He allows meat in reasonable quantity, dilute stimulants with meals so long as their use helps appetite and favours digestion. Tea if too strong, too hot, or too frequent, is liable to retard the cure of eczema, and smoking may be prejudicial. In some cases of psoriasis when associated with rheumatoid arthritis, as well as in some chronic cases of lichen planus, Morris [3] has obtained good results with exclusive meat diet and copious draughts of hot water. In acne and rosacea, indigestion and all articles of food which produce flushing must be avoided; in particular alcohol, tea, and coffee must be interdicted. In pruritus ani and vulvæ, coffee and alcohol should be proscribed; shellfish, pickles, or highly seasoned, salty, or preserved food should be avoided, and diet restricted to white meats, green vegetables, and light milk-puddings. In patients with feeble circulation showing a tendency to chilblains or Raynaud's disease carbohydrates are especially indicated. In urticaria, if obstinate, after the removal of the cause, strict milk diet in small and frequent doses may be given till the eruption disappears.

**Eczema.**—The days when arsenic was given internally for almost every skin disease have passed, but Jamieson [4] still finds it necessary to warn practitioners against its use in eczema. He says that in his experience arsenic is of little or no use in the treatment of chronic cases, while in acute forms it aggravates the disease. The association of eczema and gout or rheumatism he considers to bear no causal relationship, though the skin affection assumes a very chronic form which may call for complete rest from business anxieties, a simple open-air life, and a plain dietary. The regimen of one of the spas is to be recommended on these accounts. The chief element in the production of eczema he regards as an external one, and relies chiefly on external methods of treatment. He recommends the adoption of three cardinal principles in the external treatment: cleansing, soothing, and cautiously stimulating. These must be carried out in this order without undue haste, the effect of each procedure being watched and weighed before the next is entered upon.

**Diphtheritic Ulcers.**—It must not be forgotten that sores and wounds may be inoculated with the diphtheria bacillus. Unless the connection with faucial diphtheria were obvious, a sloughing, dirty wound due to the Klebs-Loeffler bacillus might be wrongly diagnosed. Cases of this nature which fortunately were detected are reported by Heelis [5] and Jacob. In these patients ulcers appeared on the toes, the heels, the back of the hand, and the outer canthus of the eye. They gave typical cultures of the Klebs-Lœffler bacillus, and were readily cured by painting with 10-per-cent. solution of silver nitrate.

**Pellagra.**—Pellagra is unknown, or almost unknown, in England. A case, however, though it differed in some particulars from those prevalent in Italy and the South of France, is recorded under this appellation by Andrew Cassels Brown,[6] and it bears so close a resemblance to the typical cases that his diagnosis is warranted. The patient was a girl, aged 21, who became very debilitated and complained of vague pains in the side, fingers, and ankles, and great weakness in the legs, which caused her to fall at times; sometimes she fainted. At first constipation was troublesome, but this gave place to a frequent, foul diarrhœa with salivation, ulcers on the tongue and throat, great tenderness of the abdomen and the skin generally, abnormal dryness of the skin, and a slight papular eruption, which, however, did not show the typical glazed and livid appearance usually seen. The temperature rose to over 103° F., the pulse was 126. Widal's test was negative. There was some leucocytosis, especially of the polynuclear elements, while there was apparently decrease in the lymphocytes. The mental powers were unimpaired, being rather more acute than normal. Sleep was impaired, there was slight delirium, the speech was jerky, the abdominal reflexes exaggerated, though the knee-jerks were absent; pain and tingling in the fingers suggested neuritis. The patient had been in the habit of eating maize, barley, and rye freely which composed the food she fed the poultry with. Bismuth and salol had no good result. Quinine and potassium chlorate were given, but the quinine was discontinued on a sharp uterine hæmorrhage occurring. The patient became acutely ill with a temperature of 104°, but improved later. The difference from the ordinary cases published of pellagra lies in the presence of pyrexia, neuritis, and hyperæsthesia, while the erythema noticed abroad on the parts exposed to the sun was absent in this case, perhaps because being in the winter months there was insufficient sunshine.

**Treatment of Syphilis.**—Some practical points in the administration of mercury in syphilis are recorded by Wild.[7] He urges that the drug should always be given so as to produce slight evidence of mercurialism, otherwise the desired result may not be obtained. For intramuscular injections sal alembroth is a good soluble salt. Mercury is excreted very slowly and may be detected in the urine for months after a course of the drug. Full doses of the alkaline iodides should not be given with mercury, otherwise, as Sir Wm. Gowers points out, they aid in the elimination and destroy the effect. Wild has seen cases in which mercury and pot. iodide have been administered for weeks without sign of mercurialism, but on discontinuing the iodide the same dose of mercury soon produced swelling of the gums, and improvement of the rash occurred concomitantly. In tertiary syphilis mercury is useful in preventing récurrence, and in some cases of use in the treatment of serpiginous and nodular eruptions. It should be given with caution in deep ulceration as it may cause rapid spread of the destruction of tissue. Iodides, with or without sarsaparilla, may be used in such cases. The indication for iodides is the presence of cellular infiltration, and for this purpose it is best to give full doses until this result is obtained, when a course of mercury should be given. The potassium salt is the most rapid of the iodides for diffusion. It must not be forgotten that the patient must be treated as well as the disease; iron frequently does good; quinine, strychnine, and ol. morrhuæ are extremely useful, and in tertiary cases alcohol is often of value. Opium should be given for pain, and bromides in restless nervousness. The use of sarsaparilla [8] in syphilitic cachexia is again obtaining credence, but for this purpose it must be given in large doses and frequently. Some writers advocate that the decoction must be used fresh, one or two pints being administered in the 24 hours. Clifford Allbutt considers that the decoct. sarsæ co. gives fully as good results as any other formula, ℥ss. to ℥j of the concentrated decoction, freely diluted, taken several times a day will improve nutrition and restore health.

[1] Prac., April, 1906. [2] Edin. Med. Jour., March, 1906. [3] Prac., April, 1906. [4] Edin. Med. Jour., March, 1906. [5] Brit. Med. Jour., March 10, 1906. [6] Prac., May, 1906. [7] Brit. Jour. Derm., May, 1906. [8] Brit. Med. Jour., March 24th and 31st, April 7th, and May 5th.

